# Epilepsy in Neurodegenerative Diseases: Related Drugs and Molecular Pathways

**DOI:** 10.3390/ph14101057

**Published:** 2021-10-18

**Authors:** Amanda Cano, Elena Fonseca, Miren Ettcheto, Elena Sánchez-López, Itziar de Rojas, Silvia Alonso-Lana, Xavier Morató, Eliana B. Souto, Manuel Toledo, Mercè Boada, Marta Marquié, Agustín Ruíz

**Affiliations:** 1 Ace Alzheimer Center Barcelona, Universitat Internacional de Catalunya (UIC), 08029 Barcelona, Spain; iderojas@fundacioace.org (I.d.R.); salonso@fundacioace.org (S.A.-L.); xmorato@fundacioace.org (X.M.); mboada@fundacioace.org (M.B.); mmarquie@fundacioace.org (M.M.); aruiz@fundacioace.com (A.R.); 2 Biomedical Research Networking Centre in Neurodegenerative Diseases (CIBERNED), 28031 Madrid, Spain; mirenettcheto@ub.edu (M.E.); esanchezlopez@ub.edu (E.S.-L.); 3 Department of Pharmacy, Pharmaceutical Technology and Physical Chemistry, Faculty of Pharmacy and Food Sciences, University of Barcelona, 08028 Barcelona, Spain; 4 Institute of Nanoscience and Nanotechnology (IN2UB), 08028 Barcelona, Spain; 5 Epilepsy Unit, Neurology Department, Vall d’Hebron University Hospital, 08035 Barcelona, Spain; e.fonseca@vhebron.net (E.F.); mtoledo@vhebron.net (M.T.); 6 Research Group on Status Epilepticus and Acute Seizures, Vall d’Hebron Institut de Recerca (VHIR), Vall d’Hebron Barcelona Hospital Campus, 08035 Barcelona, Spain; 7 Department of Pharmacology, Toxicology and Therapeutic Chemistry, Faculty of Pharmacy and Food Sciences, University of Barcelona, 08028 Barcelona, Spain; 8 Institute of Neurosciences (UBNeuro), University of Barcelona, 08007 Barcelona, Spain; 9 Department of Pharmaceutical Technology, Faculty of Pharmacy, University of Coimbra, 3004-531 Coimbra, Portugal; souto.eliana@gmail.com; 10 Centre of Biological Engineering (CEB), University of Minho, Campus de Gualtar, 4710-057 Braga, Portugal

**Keywords:** epilepsy, neurodegenerative diseases, Alzheimer’s disease, Parkinson’s disease, Huntington’s disease, multiple sclerosis

## Abstract

Epilepsy is a chronic disease of the central nervous system characterized by an electrical imbalance in neurons. It is the second most prevalent neurological disease, with 50 million people affected around the world, and 30% of all epilepsies do not respond to available treatments. Currently, the main hypothesis about the molecular processes that trigger epileptic seizures and promote the neurotoxic effects that lead to cell death focuses on the exacerbation of the glutamate pathway and the massive influx of Ca^2+^ into neurons by different factors. However, other mechanisms have been proposed, and most of them have also been described in other neurodegenerative diseases, such as Alzheimer’s disease, Parkinson’s disease, Huntington’s disease, or multiple sclerosis. Interestingly, and mainly because of these common molecular links and the lack of effective treatments for these diseases, some antiseizure drugs have been investigated to evaluate their therapeutic potential in these pathologies. Therefore, in this review, we thoroughly investigate the common molecular pathways between epilepsy and the major neurodegenerative diseases, examine the incidence of epilepsy in these populations, and explore the use of current and innovative antiseizure drugs in the treatment of refractory epilepsy and other neurodegenerative diseases.

## Highlights

- Epilepsy is the second most prevalent neurological disease and appears in patients with neurodegenerative diseases, thus indicating a molecular link between them;
- There is growing evidence that relates the appearance of β-amyloid plaques, neurofibrillary tangles, α-synuclein, or mutations in the huntingtin protein to increased neuronal excitability that precedes seizures;
- Several approved drugs, such as atorvastatin, ceftriaxone, losartan, anakinra, rapamycin, and fingolimod, have been studied in animal models for antiseizure applications;
- Commonly used antiseizure drugs, such as levetiracetam, zonisamide, and valproate, are being investigated in other neurodegenerative diseases.

## 1. Introduction

Epilepsy is a chronic disease of the central nervous system (CNS) characterized by an imbalance in neuronal electrical activity, which leads to various recurrent and unpredictable seizures [1]. Some epileptic syndromes have been related to progressive cortical thinning and brain volume loss, as well as to neuronal death in several brain regions [2,3]. According to the latest Global Burden of Disease study, epilepsy is considered the second most serious neurological disease in the world in terms of disability-adjusted life years [4]. In 2016, it was estimated that there were 45.9 million people with all-active forms of epilepsy worldwide, with an age-standardized mortality rate of 1.74 per 100,000 individuals [4]. Globally, it is estimated that 2.4 million people are diagnosed with epilepsy each year. According to the Brainstorm Consortium, epilepsy is the most heritable neurological condition [5]. In developed countries, there are between 30 and 50 new cases per year per 100,000 people in the general population. In contrast, in developing or underdeveloped countries, this figure can be up to two times higher. This is due to the increased risk of endemic diseases, birth-related injuries, variations in medical infrastructure, and the low availability of preventive health programs [6]. A meta-analysis carried out by Fiest et al. pointed out that the lifetime prevalence of epilepsy is 7.60 per 1000 people worldwide, encompassing epilepsies of unknown etiology and those with generalized seizures, which have a higher prevalence [7].

Seizures are the result of bursts of abnormally excessive or synchronous neuronal activity in the brain that can cause a wide range of symptoms. Seizures can involve a specific brain area or network (focal-onset seizures) or a synchronic bihemispheric discharge (generalized-onset seizures) [8]. Epilepsy classification is complex and includes different levels, from seizure types to epilepsy syndromes, which encompass several clinical features, such as age of onset, specific etiologies, and comorbidities [8]. According to the latest International League Against Epilepsy (ILAE) classification, epilepsy etiologies can be classified into structural, genetic, infectious, metabolic, immune, or unknown etiologies [8].

**Figure 1:**
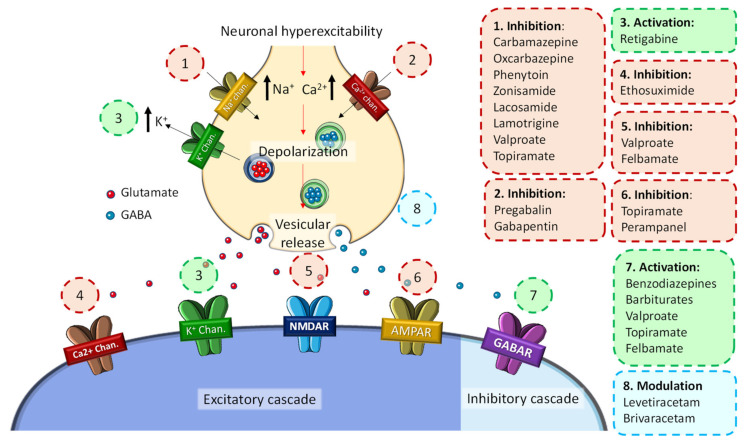
General molecular mechanisms of the development of seizure activity in epilepsy and associated ASDs.

At the molecular level, these disorders promote the depolarization of the presynaptic membrane, which has been described as the main cause of neuronal hyperexcitability that triggers the abnormal electrical activity characteristic of epileptic seizures (Figure 1). Hyperstimulation causes a conformational change in several ion channels and membrane receptors, which leads to a massive flow of Ca^2+^ and/or Na^+^ ions into the neuron and an outflow of K^+^ ions. In turn, this ionic imbalance causes the activation of different signaling cascades that promote neurotoxic effects and neuronal plasticity changes, ultimately leading to cell death [9].

Since the late 19th century, when Hughlings Jackson proposed that seizures were due to focal neuronal firing, the cerebral cortex has been considered the predominant anatomical source of seizures [10,11,12]. In recent years, the findings of histopathological, electrophysiological, and quantitative neuroimaging studies have provided ample evidence demonstrating that both focal- and generalized-onset seizures involve diverse interactions between neural networks of cortical and subcortical structures [13]. Likewise, it has been described that seizures are due not only to generalized alterations between different brain structures but also dysfunctional neural networks dominated by excessive or hypersynchronous paroxysmal activity [13]. Focal epilepsy is the most common type of epilepsy in adults, in which the main area of seizure initiation is the temporal lobe, although foci of origin have also been observed in the frontal, parietal, and occipital lobes (in descending order of frequency) [14]. Likewise, the amygdala-hippocampal complex is one of the key anatomical circuits involved in the epileptogenic process. Hippocampal sclerosis represents the paradigmatic histological finding and representative form of neuronal loss in temporal lobe epilepsy [15].

Because of the many types of epilepsy syndromes and their different causes, epileptogenic foci, and manifestations, the therapeutic approach to epilepsy is also complex and, in many cases, ineffective [16]. At the beginning of the 20th century, the first antiepileptic drugs appeared (e.g., phenobarbital, valproate, benzodiazepines), and it was not until the 1990s that the second-generation drugs (e.g., gabapentin, pregabalin, lamotrigine, levetiracetam, topiramate) emerged as new treatment options in the clinical practice [17]. Because of that, in recent years, third-generation drugs (e.g., lacosamide, rufinamide, perampanel) have emerged. These substances possess an enhanced controlled central activity and a more favorable pharmacokinetic profile (Figure 1) [17]. However, these medications are focused almost exclusively on seizure control and not on the epileptogenic mechanisms, which is why they are currently referred to as antiseizure drugs (ASDs) [17]. Therefore, in this review, we thoroughly investigate the common molecular pathways between epilepsy and the major neurodegenerative diseases, examine the incidence of epilepsy in these populations, and explore the use of current and innovative ASDs in the treatment of refractory epilepsy and other neurodegenerative diseases.

## 2. Epilepsy in Neurodegenerative Diseases

### 2.1. Epilepsy and Alzheimer’s Disease

Alzheimer’s disease (AD) is the most common form of dementia, affecting 50 million people worldwide, and is characterized by memory loss and cognitive decline associated with neurodegenerative processes [18]. The main hypothesis for the neurotoxicity and synaptic dysfunction in AD focuses on the typical pathological hallmarks of the disease, mainly intracellular neurofibrillary tangles (NFTs) of phosphorylated tau (p-tau) and extracellular amyloid-β (Aβ) senile plaques, although many other mechanisms involved in AD pathogenesis have been described [19].

Regarding the frequency of epilepsy in AD patients, it has been reported that individuals suffering from AD have a more than 80 times higher risk of developing seizures compared to individuals without AD [20]. On the other hand, patients with epilepsy have a higher risk of developing dementia over the years [21]. For these reasons, it has been argued that the increased incidence of seizures in AD could just be due to the fact that the onset of AD typically occurs after the age of 65 [22]. However, the relationship between epilepsy and AD has created much controversy. Whereas several studies have shown a higher incidence of seizures among AD patients, many authors have highlighted that, according to the type of monitoring used and the population studied, the prevalence of seizures in AD may range from 3.5% to 64% [23].

The molecular relationship between the pathological mechanisms of AD and epilepsy has been widely studied because of the evidence of common, pervasive brain glucose hypometabolism, spatial memory and navigation deficits, damage in hippocampal neurons, and general neurodegeneration in the temporal lobe [20]. Interestingly, senile plaques were first described in epileptic patients more than 10 years earlier than the first report of a case of AD [24]. In fact, the first clinical studies that evaluated the relationship between AD and epilepsy date back to the early 1950s [25,26]. Both diseases involve neuronal damage and also appear to have a bidirectional association [27].

The research group of Dr. Cole carried out an interesting study in the Epilepsy Service of Massachusetts General Hospital and Harvard Medical School in Boston. They found that patients with AD experienced subclinical seizures during sleep without clinical manifestations. This study highlighted the hypothesis that seizures might modulate, promote, or accelerate the pathological pace of AD [28]. Similarly, a study performed 10 years ago found that 42% of AD patients developed subclinical seizures, compared to 11% in the control group. This epileptic activity originated mainly in the temporal lobe during the deeper stages of sleep. Moreover, over a 5-year period, these AD patients showed increased cognitive decline compared to AD controls without subclinical seizures [20].

**Figure 2:**
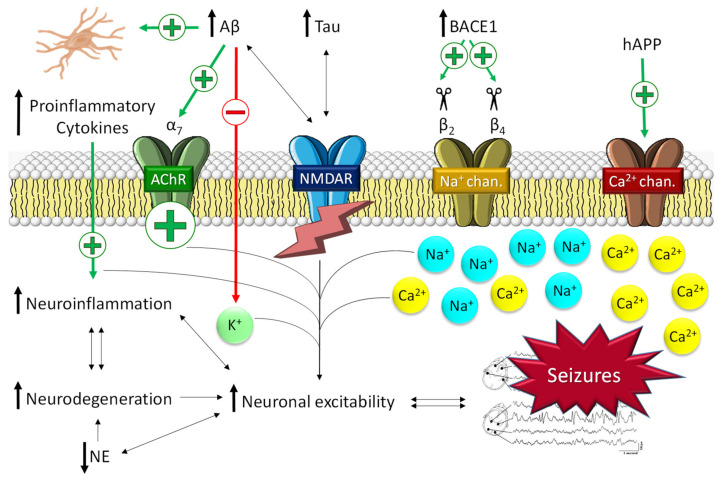
Seizure activity derived from the main pathological molecular pathways of Alzheimer’s disease. The pathological hallmarks of Alzheimer’s disease promote an increase in neuroinflammation and intracellular Ca^2+^ through ACh and NMDA receptors and Na^+^/Ca^2+^ channels. This promotes an increase in neuroinflammation and neuronal hyperexcitability, which in turn increases the neurodegeneration process (and *vice versa*) in a vicious cycle. NE, norepinephrine.

Several mechanisms connecting epilepsy and AD have been described. Recent experimental data suggest that neuronal hyperexcitability itself might play an important role in promoting the neuropathological burden and cognitive decline of AD [22]. Thus, the increase in amyloid-β (Aβ) and tau peptide levels characteristic of AD has been related to the molecular pathways that trigger seizures (Figure 2).

#### 2.1.1. The Role of Aβ in Epilepsy

Patients affected by hereditary AD, which is typically caused by mutations in the amyloid precursor protein (APP), presenilin-1 (PS1), and/or presenilin-2 (PS2) genes, are a particularly seizure-prone population, with seizures rates higher than 30% [29]. These findings support the key role of Aβ in epileptic susceptibility (Figure 2). In addition, a derived hypothesis describes a vicious cycle in which AD molecular alterations promote seizures [22], which in turn may exacerbate AD pathology [29]. In AD, soluble oligomeric Aβ, rather than Aβ plaques, has been reported to be the main cause of neuronal hyperexcitability [22]. Thus, Aβ_1-42_, the most toxic form of Aβ soluble peptides, has been found to increase neuronal excitability by selectively inhibiting K^+^ currents [30]. Glutamate signaling has also been described to be altered by Aβ in AD patients. The impairment of neuronal and glial glutamate reuptake may lead to glutamate spillover and, consequently, excitotoxicity. Likewise, glutamate excitotoxicity is also exacerbated by the effect of Aβ on N-methyl-D-aspartate receptor (NMDA-R) trafficking [31]. Kam et al. hypothesized that the activation of cholinergic receptors and Ca^2+^ channels by Aβ might trigger early subclinical epileptic activity preceding clinical AD [32]. Indirectly, beta-secretase 1 (BACE1), one of the main proteins involved in the formation of Aβ, has also been related to the promotion of epileptogenic processes (Figure 2) [22]. Several studies have described that BACE1 cleaves the β_2_ and β_4_ subunits of the voltage-gated Na^+^ channel. β_2_ cleavage alters the transcription and expression of the receptor on the cell surface [33]; β_4_ cleavage significantly increases the intracellular levels of Na^+^ [34]. Both processes lead to general neuronal hyperexcitability that ultimately conduces to the development of seizures. In preclinical studies, Kim et al. demonstrated the physiological changes in sodium channel metabolism in BACE1-null mice [35]. They found that Na_v_1.1 protein levels and Na_v_β_2_ processing were significantly decreased in BACE1-null versus wild-type mouse brains. Interestingly, hippocampal surface Na_v_1.1 levels were significantly decreased, but Na_v_1.2 surface levels were increased in BACE1-null mice, perhaps as a compensatory mechanism for reduced surface Na_v_1.1 levels. All these results caution that therapeutic inhibition of BACE1 activity may affect Na^+^ metabolism and alter neuronal membrane excitability in AD patients [35]. Likewise, it has been described that BACE inhibitors might be involved in the development of seizures. In that respect, it has recently been reported that BACE inhibitors can induce hyperactivity in persons carrying a seizure-related gene family without altering learning and memory [36].

The potential causative role of Aβ in the development of neuroinflammation and, in turn, the generation of seizures has also been described (Figure 2). Neuroinflammation is characterized by the induction of an immune reaction in the CNS as a response to a pathological process and has been detected in both epilepsy and AD [37]. Inflammation in the CNS is mediated mostly by microglia, astrocytes, and oligodendrocytes [38]. The glial activation by Aβ leads to the release of numerous proinflammatory cytokines (i.e., TNF-α, IL-6, or IL-1β), giving rise to the appearance of generalized neuroinflammation. This process, in turn, promotes neurotoxic effects, which ultimately lead to the appearance of neuronal hyperexcitability, in turn increasing the neurodegeneration process in a vicious cycle [22]. Likewise, proinflammatory cytokines, such as IL-1β, have been described to increase neuronal hyperexcitability by enhancing glutamate release by astrocytes and reducing its reuptake [39] or by upregulating NMDA-Rs, which increases the intracellular Ca^2+^ influx [40]. Moreover, in vivo and in vitro studies have provided evidence for a bidirectional relationship between exacerbated inflammation and seizures; both events feed back into each other in a vicious circle [39].

#### 2.1.2. The Role of Tau in Epilepsy

Animal models have been very useful in understanding the role of tau in the generation of seizures (Figure 2). A preclinical model of transgenic APP/knock-out tau mice suggested that tau protein is a necessary mediator of the epileptogenic effects of Aβ [41]. In this study, transgenic mice exhibited less frequent and less severe seizures than wild-type mice. Tau protein has also been shown to promote marked neuronal excitotoxicity by increasing extracellular glutamate and NMDA-R dysfunction [42]. Likewise, tau has also been related to abnormal neuronal migration in the hippocampus, which is closely involved in epilepsy development [43].

In 2011, a postmortem study in patients with chronic epilepsy revealed that almost 70% of the analyzed brains exhibited mild or moderate AD tau pathology [44]. Tau burden was significantly related to progressive cognitive decline, with focal epilepsy being more often associated with higher tau burden in patients with chronic epilepsy than in patients with idiopathic or genetic generalized epilepsy [44]. Likewise, a study in three different animal models of epileptogenesis found a decrease in phosphatase 2A activity, the enzyme responsible for phosphorylation/dephosphorylation within cells, which led to an increase in p-tau in the epileptogenic brain regions [45].

#### 2.1.3. The Role of Allopregnanolone in AD and Epilepsy

Allopregnanolone is a naturally occurring neurosteroid derived from the hormone progesterone. Accumulating evidence points toward a molecular relation between allopregnanolone and AD development [46]. Several authors have reported reduced plasma and brain levels of allopregnanolone in the prefrontal cortex of AD patients [46]. Curiously, Luchetti et al. reported increased levels of the mRNA levels of the enzyme aldoketoreductase C2, which leads to the synthesis of allopregnanolone in the brains of the early AD neuropathological stage [47]. It has been hypothesized that this increase is a compensatory mechanism of the prefrontal cortex to raise the levels of allopregnanolone, but further studies would be necessary to fully understand this event. Declining allopregnanolone levels, as well as other neurosteroids, have been suggested to lead to reduced neuroprotection. This could indeed be one of the bases for increased apoptosis and neuronal cell loss, which may therefore contribute to neurodegenerative processes and hyperexcitability, which finally lead to the appearance of seizures. Likewise, it has been also described that the reduced levels of allopregnanolone may chronically activate the astrocytes and microglia [46]. This activated microglia around the plaques, have been promote the production of neurotoxic cytokines, chemokines, and reactive oxygen and nitrogen species, which also contribute to the increase in neuronal excitability and finally seizures.

### 2.2. Epilepsy and Parkinson’s Disease

Parkinson’s disease (PD) is a neurodegenerative disease characterized by a progressive loss of dopaminergic nerve endings in the substantia nigra and striatum, which leads to motor and coordination symptoms but also to cognitive decline, depression, and anxiety [48]. PD is the second most prevalent neurodegenerative disease and the most common motor disorder [49]. The origin of PD is not yet clear, but it has been hypothesized that it may involve mutations in specific genes and environmental causes [48]. PD patients exhibit a reduced dopaminergic activity and alterations in the structure of α-synuclein, a presynaptic protein that seems to play an important role in the development of PD [50]. Dopaminergic neurons can become damaged as a result of the toxicity of oligomeric forms of α-synuclein, endoplasmic reticulum (ER) stress, autophagy processes, dysfunction of calcium homeostasis, and changes in the function and structure of mitochondria [51]. α-synuclein is also the main component of Lewy bodies, which are closely related to PD dementia and have been found in the locus coeruleus of more than 50% of PD patients [52]. The misfolding and aggregation of α-synuclein commonly appear in the development of sporadic PD. Some authors have reported that these aggregates might be able to propagate transsynaptically from cell to cell, from the enteric nervous system or olfactory bulb all the way to the cerebral cortex, although the transsynaptic movement of α-synuclein has not been conclusively demonstrated in these type of neurons [53].

Although typical symptoms involve tremor, rigidity, or bradykinesia, prototypic PD and other forms of Parkinsonism can also show epileptic seizures and status epilepticus [54]. According to the Brainstorm Consortium, there is no genetic correlation between PD and epilepsy [5]. Existing observational studies of the incidence of epileptic seizures in PD patients are based on cross-sectional data, small and heterogeneous study populations, or data that were not adjusted for confounding factors. However, Feddersen et al. reported that 2.6% of PD patients develop epilepsy [54]. This value is very similar to that reported by Bodenmann et al. 20 years ago, showing a prevalence of 2.4% [55]. A retrospective cohort study with a nested case-control analysis recently conducted by Gruntz et al. revealed that, among 23,086 patients with incident PD and 92,343 PD-free individuals, 898 patients were identified with incident epileptic seizures [56]. The number of people who suffered from epileptic seizures in the PD patients’ cohort was twice as large as that in the PD-free cohort, being 266.7/100,000 and 112.4/100,000 person-years, respectively. In addition, the adjusted odds ratio (OR) of epileptic seizures was 1.68 in PD patients compared with PD-free individuals. Likewise, PD patients with more than one seizure-provoking comorbidity were at the highest risk of epileptic seizures compared with PD-free individuals with no seizure-provoking comorbidities. This study clearly suggests that incident PD is associated with an increased risk of incident epileptic seizures [56]. However, this study did not reveal whether these findings were due to differences at the molecular level, concomitant drugs taken by the study’s patients, or the degree of causality. Thus, further studies are needed to clarify these issues.

Regarding the available treatments, it is important to highlight that many drugs for PD possess antiepileptic properties, such as L-DOPA or apomorphine, which could alter the real values of the cross-sectional prevalence between both diseases [57,58].

#### 2.2.1. The Role of α-Synuclein in Epilepsy

**Figure 3:**
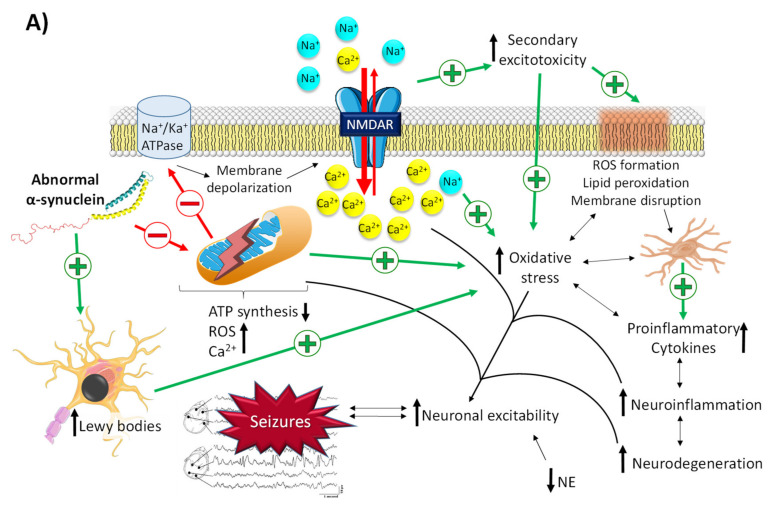

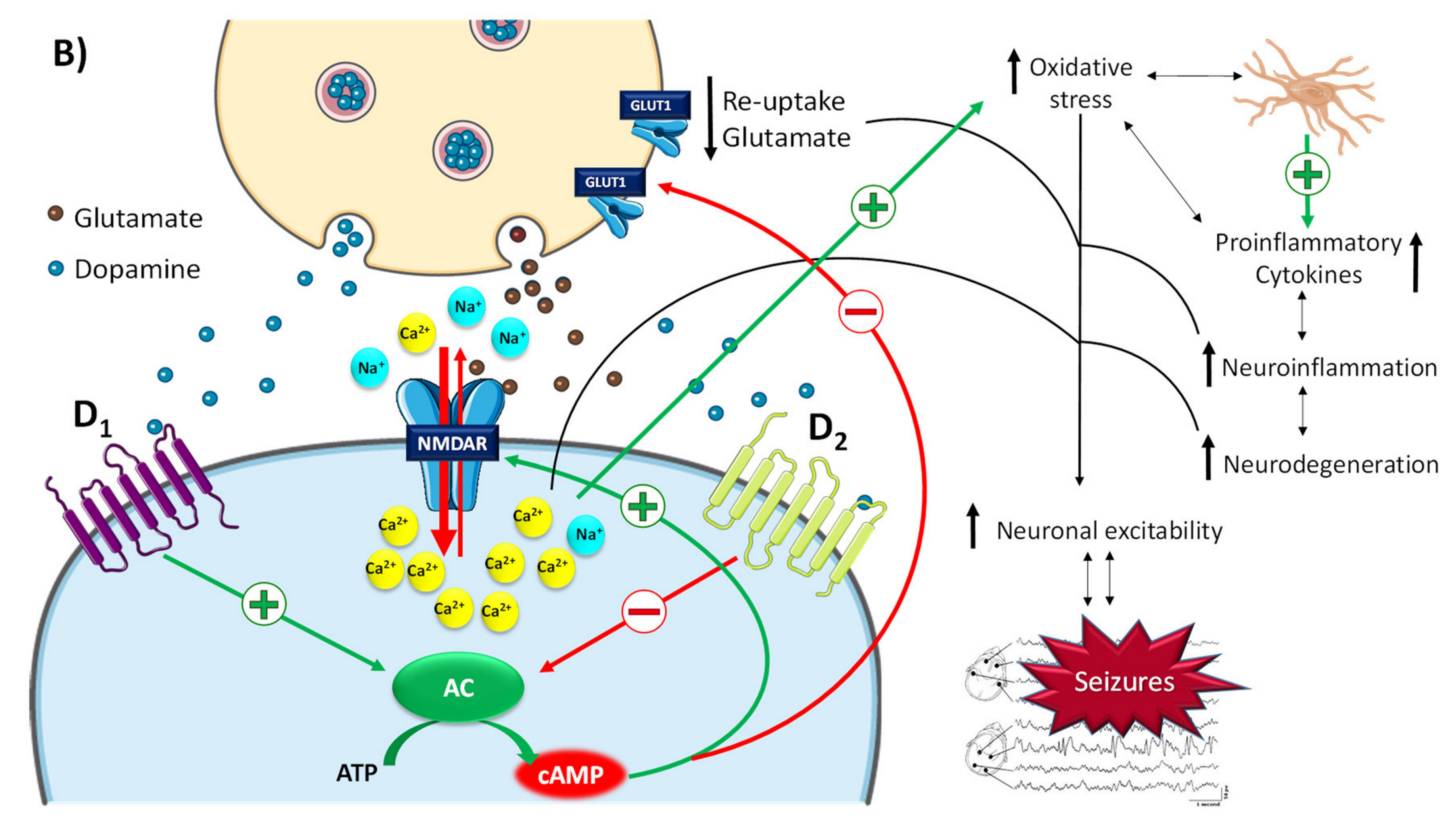
Related molecular pathways between Parkinson’s disease and epilepsy. (**A**) Neuronal excitability via mitochondrial dysfunction derived from the accumulation of abnormal α-synuclein. Abnormal α-synuclein promotes membrane depolarization, massive influx of intracellular Ca^2+^, and oxidative stress through the induction of mitochondrial dysfunction and Lewy bodies’ formation. This promotes an increase in neuroinflammation and neuronal hyperexcitability, which in turn increases the neurodegeneration process (and *vice versa*) in a vicious cycle. (**B**) Proepileptic/antiepileptic properties of dopamine conditioned by its binding to the D_1_/D_2_ family of receptors. Binding of dopamine to D_1_R promotes an increase in cAMP, which leads to the activation of NMDA-Rs and blockage of GLUT1, thus promoting a massive influx of intracellular Ca^2+^ and a reduction in glutamate reuptake. This gives rise to an increase in neuroinflammation and neuronal hyperexcitability, which in turn increases the neurodegeneration process (and *vice versa*) in a vicious cycle. Binding of dopamine to D_2_R inhibits the production of cAMP, thus promoting the opposite effect of that of D_1_R activation. NE, norepinephrine; ROS, reactive oxygen species.

The role of α-synuclein in the pathophysiological mechanisms that trigger PD and epileptogenic events is closely related to mitochondrial dysfunction (Figure 3A) [51,59,60]. As described above, the accumulation of misfolded α-synuclein leads to the formation of Lewy bodies in susceptible neurons, located mainly in the basal ganglia. Likewise, abnormal α-synuclein has also been described to affect the structure of mitochondria at different levels [51]: (i) alterations in voltage-dependent anion channels located in the mitochondrial membrane, which are involved in calcium transport between the endoplasmatic reticulum and the mitochondria, resulting in a massive entrance of Ca^2+^ and, consequently, organellar hyperexcitability that provokes mitochondrial dysfunction; (ii) disruption of protein import through the outer mitochondrial membrane by binding to the TOM22 receptor, which results in a decrease in the activity of complex I, depolarization of mitochondria, dysregulation of Ca^2+^ homeostasis, and overproduction of reactive oxygen species (ROS); (iii) direct inhibition of complexes I and V of the electron transport chain of mitochondria; (iv) mitochondrial depolarization, whose consequence is the accumulation of the serine/threonine kinase PINK1 in the mitochondrial outer membrane, which in turn initiates the removal of damaged mitochondria by autophagy; and (v) inhibition of mitochondrial sirtuin 3, an enzyme that plays a key role in the prevention of oxidative stress and the maintenance of mitochondrial function and whose inhibition contributes to impaired mitochondrial biogenesis and dynamics [51,60].

Both mitochondrial dysfunction and Lewy bodies are the triggers for a vicious circle in which there is an increase in ROS levels and oxidative stress, peroxidation of membrane lipids that enhances membrane disruption, activation of glia, and the release of proinflammatory cytokines, leading to an increase in neuroinflammation, neurodegeneration, and, ultimately, neuronal hyperexcitability (Figure 3A) [53].

#### 2.2.2. The Role of Dopamine and Norepinephrine in Epilepsy

As mentioned above, dopamine has been described to possess antiepileptic activity. However, this effect is conditioned by the family of receptors it binds to [53]. There are two families of dopamine receptors: the D_1_ family, which comprises D_1_ and D_5_ dopamine receptors, and the D_2_ family, which comprises D_2_, D_3_, and D_4_ receptors. When dopamine binds to both subtypes, the effect is opposite [53]: the activation of D_1_-like receptors enhances the activation of adenyl cyclase, which produces an increase in cAMP and thus leads to the activation of NMDA-Rs and blockage of GLUT1. All this results in an increase in glutamate, intracellular Ca^2+^, oxidative stress, and proinflammatory cytokines, stimulating neuronal hyperexcitability and leading to seizures (Figure 3B) [61]. With regard to that, a study performed in the 90s already showed that the activation of D_1_ receptors in patients with refractory epilepsy promoted the development of epileptic activity by increasing cortical excitability, whereas D_2_ receptor agonists exhibited the opposite effect [62].

Postmortem brain analysis of well-established PD patients showed a widespread reduction in the levels of the neurotransmitters norepinephrine, acetylcholine, and serotonin, with norepinephrine being the most affected [63]. The neuronal network of the locus coeruleus was the most affected, as most of the norepinephrine neuronal circuit lies there. Interestingly, most of the Lewy bodies’ accumulation also appears in this brain region [53]. This reduction might be associated not only with PD-related depression but also with the appearance of epileptic activity since norepinephrine modulates neuronal excitability [64]. In preclinical studies, animals with lesions of the noradrenergic system are more vulnerable to hippocampal kindling and seizures [65]. However, whether these statements also apply to humans is not completely clear, so more studies are needed to confirm this hypothesis.

#### 2.2.3. The Role of Allopregnanolone in PD and Epilepsy

There are some studies that have analyzed alterations of neurosteroid levels in PD patients. Bixo et al. found 20 years ago increased levels of allopregnanolone in the substantia nigra and caudate nucleus of control subjects, indicating that synthesis of this neurosteroid takes place in the dopaminergic system [66]. By contrast, in PD patients, di Michele et al. reported reduced levels of allopregnanolone in the cerebrospinal fluid, thus suggesting a molecular link for progesterone metabolites in this disease [67]. Moreover, the mRNA expression of two enzymes that synthesize allopregnanolone, 5α- reductase type 1 (SRD5A1) and aldoketoreductase C3 (AKR1C3), was found to be significantly reduced in peripheral blood mononuclear cells of PD patients [46]. This suggests a generalized defect in the enzymatic machinery that regulates the metabolism of progesterone. Likewise, SRD5A1 was downregulated in the substantia nigra, which, interestingly, was mirrored by upregulation of AKR1C2 in the caudate nucleus, suggesting involvement of allopregnanolone in the neurodegenerative process [46]. All these facts would be related to the reduction in neuroprotection and the increase in neuronal excitability, which finally lead to seizure development. However, further studies in large cohorts of patients are needed to validate all these findings.

### 2.3. Epilepsy and Huntington’s Disease

Huntington’s disease (HD) is a rare, autosomal-dominant neurodegenerative disease that involves motor dysfunction, incoordination, chorea and dystonia, behavioral difficulties, and cognitive decline [68]. Just as in PD, the caudoputamen and basal ganglia are the most affected areas in HD. HD is triggered by a mutation in the huntingtin (HTT) gene, which leads to the overproduction of misfolded huntingtin protein (mHtt) [69]. In exon 1 of chromosome 4, the mutated gene exhibits a pathogenic genomic expansion of the CAG trinucleotide repeat. In general, the greater the number of CAG repeats, the earlier the onset of HD [70].

Early-onset HD (also called juvenile HD) is very rare (less than 10% of cases), associates preferentially with paternal transmission, and presents a severe and rapid disease progression [53,71]. In this cohort of patients, particularly in childhood-onset HD, epileptic phenomena are common, whereas, in adult-onset HD, they rarely occur [53,71]. The most common seizure types in HD patients that have been documented are generalized tonic-clonic and myoclonic seizures, suggesting that cortical and limbic structures are involved [53]. There is not much information available regarding the incidence of epilepsy in HD. A study performed by Cloud et al. in juvenile HD patients showed that seizures were present in 38% of subjects [72]. Generalized tonic-clonic seizures were the most common seizure type, followed by tonic seizures, myoclonic seizures, and staring spells. Furthermore, they found that seizure risk increases with younger age at HD onset. Conversely, Spila et al. studied the frequency of epileptic seizures in adult-onset HD patients and reported that the prevalence of epilepsy in patients with adult-onset HD was similar to that in the general population [73]. However, the retrospective nature of these studies limited their ability to obtain conclusive results. Future prospective studies with more patients enrolled are therefore needed to validate all these findings.

#### 2.3.1. The Role of mHtt in Epilepsy

**Figure 4:**
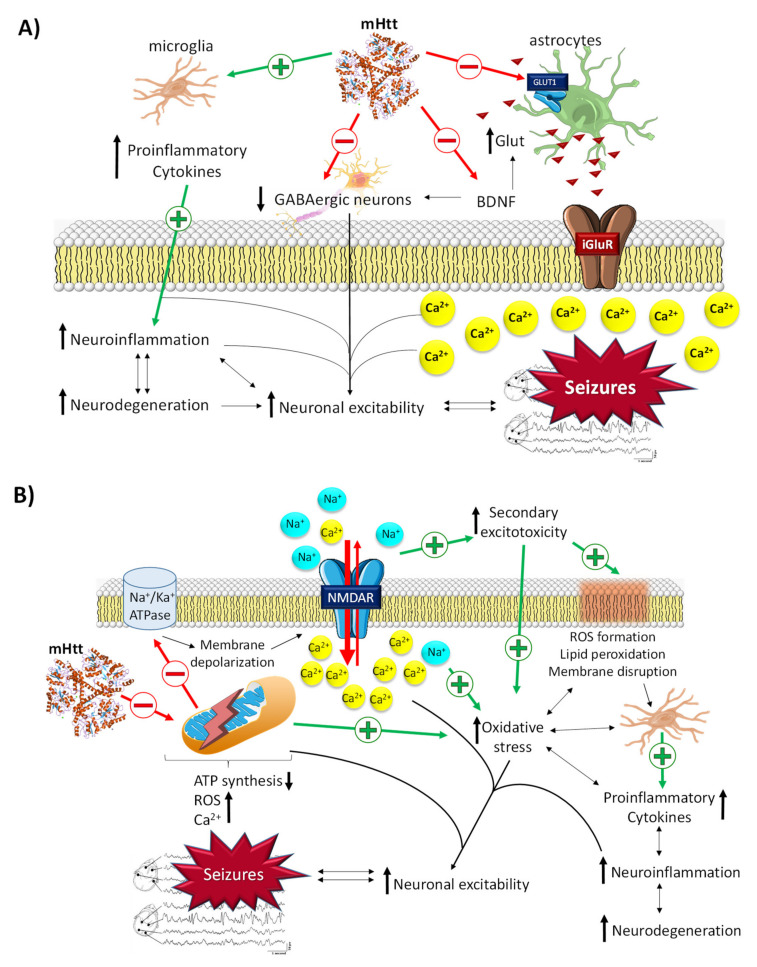
Related molecular pathways between Huntington’s disease and epilepsy. (**A**) General mechanisms by which mHtt leads to the development of seizures. (**B**) Neuronal excitability via mitochondrial dysfunction derived from the damage promoted by mHtt. mHtt promotes membrane depolarization, massive influx of intracellular Ca^2+^, and oxidative stress through the induction of mitochondrial dysfunction and microglia activation and the inhibition of astrocyte GLUT1Rs, BDNF, and GABAergic neurons. All this promotes an increase in neuroinflammation and neuronal hyperexcitability, which in turn increases the neurodegeneration process (and *vice versa*) in a vicious cycle.

Although the HTT gene mutation was described by Gusella et al. in 1983 [74], the role of mHtt in the onset and progression of HD is not yet well known. In epilepsy, mHtt has been described to contribute to neuronal hyperexcitability by different mechanisms (Figure 4A,B) [53]. mHtt possesses a dual action on glial cells. On the one hand, it activates microglia, which leads to a massive secretion of proinflammatory cytokines, an increase in neuroinflammation, neurodegeneration, and, finally, neuronal hyperexcitability [75]. On the other hand, it impairs glutamate uptake by damaging the GLUT1 transporters of the membrane of astrocytes. This results in an increase in glutamate in the synaptic space, which causes the excitotoxic cascade typical of this neurotransmitter [75]. Likewise, mHtt has been reported to promote transcriptional dysregulation of essential genes, such as the gene for brain-derived neurotrophic factor (BDNF), which leads to neuronal hyperexcitability through the enhancement of glutamatergic responses and the inhibition of GABAergic responses [76]. Emerging evidence also suggests that mHtt alters mitochondrial function, which triggers defective Ca^2+^ homeostasis, aberrant ROS production, an alteration in mitochondrial protein import, an increase in mitochondrial fragmentation, and, finally, a decrease in ATP production [75]. As in PD, these mitochondrial alterations give rise to several cascades of excitotoxic molecules that cause seizure activity in epilepsy.

#### 2.3.2. The Role of BDNF in Epilepsy

In HD, reduced levels of BDNF and impaired function of receptors with high affinity to this protein (TrkB) have been reported [76,77]. These alterations have been related to reduced neuronal gene transcription of both BDNF and TrkB caused mainly by mHtt [53]. However, the role of BDNF in epilepsy is highly complex. Although some authors have mentioned the protective effects of BDNF against excitotoxicity produced during seizures, BDNF’s contribution appears to be mostly proepileptic [53]. Studies performed in the 90s already reported that a significant increase in BDNF decreases the responses of GABAergic neurons and increases the levels of interstitial glutamate, thereby directly promoting neuronal hyperexcitability (Figure 4A) [78,79]. By contrast, other studies suggest that sustained levels of BDNF could promote antiepileptic effects via the NPY peptide, which has been shown to possess clear antiepileptic activity [80]. Interestingly, NPY/somatostatin interneurons are increased in HD patients, thus suggesting the existence of compensatory mechanisms before the cerebral cortex becomes hyperexcitable in these patients [53]. Furthermore, hippocampal BDNF expression has been shown to have potential positive effects on cognitive performance in post-status epilepticus rat models [81]. Likewise, it has been reported that BDNF has a protective role in neurodegeneration through its antiapoptosis and antioxidant effects and suppression of autophagy [82]. These results raise the possibility of a molecular target for the treatment of epileptogenesis, although it is unknown whether the cognitive effects are derived directly from BDNF signaling or are secondary to the suppression of critical activity. On the other hand, epileptogenic models in which BDNF signaling has been tested are mostly based on epilepsies of structural origin, and whether these signaling pathways are shared in different etiologies remains a matter of debate.

### 2.4. Epilepsy and Multiple Sclerosis

Multiple sclerosis (MS) is a heterogeneous and complex autoimmune disease of the CNS characterized by demyelinating processes and axonal damage. It affects more than 2 million people around the world and is considered the most prevalent chronic inflammatory disease of the CNS [83]. Although MS is not categorized as a purely neurodegenerative disease, its typical pathological processes lead to prolonged and irreversible destruction of neural tissue [84,85,86].

Although the causes of its pathogenesis are not entirely clear, it is known that MS development is associated with a combination of genetic and environmental factors. Interestingly, genetic data suggest that the pathogenesis of MS shares important features with a variety of non-CNS autoimmune diseases [83,87]. Moreover, the existence of an increased intestinal permeability has also been highlighted as a potential cause of MS. This alteration would allow the uncontrolled passage of substances into the blood (e.g., viruses, bacteria, toxins), which could cause an abnormal response of the immune system [88].

**Figure 5:**
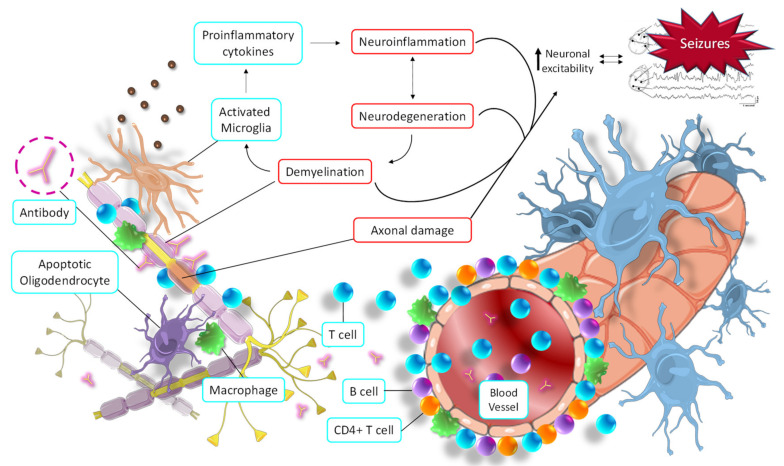
Seizure activity derived from the main pathological molecular pathways of multiple sclerosis. Autoimmune responses promote demyelination and axonal injury, which in turn trigger the activation of microglia, oligodendrocytes, and macrophages, thus initiating neuroinflammation and neurodegeneration. All this increases neuronal hyperexcitability, which in turn increases the neurodegeneration process (and *vice versa*) in a vicious cycle.

MS lesions can appear throughout the CNS and are most easily recognized in the white matter as focal areas of demyelination, inflammation, and glial reaction. Tissue damage in MS results from a complex and dynamic interplay between the immune system, glia (myelin-making oligodendrocytes and their precursors, microglia, and astrocytes), and neurons. The cells involved in autoimmune inflammatory damage in MS are mainly lymphocytes (T and B lymphocytes), macrophages, and microglia. In MS patients, the blood-brain barrier (BBB) is damaged, allowing autoreactive T lymphocytes to pass. Inside the brain, these T cells destroy the myelin sheaths, and surrounding inflammation is facilitated by other immune cells and soluble elements, such as cytokines and antibodies (Figure 5) [88].

The clinical manifestations of MS are very heterogeneous. It typically presents as a sensory and/or motor disorder, optic neuritis, fatigue, trigeminal neuralgia, or vertigo [89]. As with PD, the Brainstorm Consortium reported that there is no genetic correlation between MS and epilepsy [5]. However, seizures can appear in MS patients [90]. Given the anatomical variability of demyelinating lesions, a wide variety of seizure types has been observed in MS patients [91]. A retrospective study carried out on 310 patients with MS reported that 3.2% suffer from epilepsy. In these patients, seizures were the first MS symptom, and the most frequent seizure type was partial secondary generalized seizures. Furthermore, these patients were younger and had an earlier onset of MS symptoms compared to the group without epilepsy, and all showed cortical lesions [92].

Although the molecular link between epilepsy and MS has not been fully clarified, some hypotheses have been put forward. The autoimmune hyperactivity that causes the demyelinating process leads to the activation of both astrocytes and microglia, as well as the initiation of the apoptosis process of oligodendrocytes [93]. All these mechanisms cause a massive release of proinflammatory cytokines and a general increase in CNS inflammation. In turn, this promotes the neurodegeneration process and stimulates the demyelinating process, causing a vicious cycle of destruction of neural tissue. These pathophysiological alterations contribute to increased neuronal hyperexcitability, the main cause of the development of seizures (Figure 5) [94]. Likewise, direct axonal damage caused by antibodies, T lymphocytes, proinflammatory cytokines, macrophages, etc., also directly contributes to an imbalance in the electrical activity of neurons. This alteration affects the membrane potential oscillations in neurons, leading to their hyperexcitability and, finally, seizures (Figure 5) [94].

#### The Role of Allopregnanolone in MS and Epilepsy

Some studies have reported that allopregnanolone may target common pathological pathways in MS and epilepsy [95]. Regarding MS, it has been reported that an impaired neurosteroid synthesis in multiple sclerosis [96]. In this sense, Noorbakhsh et al. showed that the administration of allopregnanolone in mice with autoimmune demyelination ameliorated the neurobehavioral deficits and improved the neuropathology and neuroinflammation [97]. The same authors showed that levels of several neurosteroids, including allopregnanolone, were suppressed in the white matter of patients with MS [96]. Regarding epilepsy, Meletti et al. identified allopregnanolone as a positive modulator of inhibitory currents mediated by GABA-A receptors in epilepsy. Similarly, Lévesque et al. showed an effect of allopregnanolone in modulating ictogenesis and the occurrence of hyperexcitatory neuronal activity. Furthermore, they also demonstrated that allopregnanolone treatment delayed the onset of spontaneous seizures in animal models of mesial temporal lobe epilepsy [98].

## 3. Current Standards for Epilepsy Treatment and Refractory Epilepsy

The first ASDs were discovered serendipitously in the late 19th century. Years later, the use of animal models allowed the development of different molecules and their derivatives, and currently, a wide variety of drugs are available to prevent the occurrence of seizures in patients with epilepsy.

According to their main mechanism of action, ASDs can be classified into four broad categories: (i) modulation of voltage-gated ion channels, (ii) enhancement of GABA-mediated inhibitory neurotransmission, (iii) attenuation of glutamate-mediated excitatory neurotransmission, and (iv) modulation of neurotransmitter release via presynaptic action. Some ASDs have combined mechanisms of action, and in some cases, they are not fully understood (Table 1) [99,100]. Despite this wide variety of treatments, one-third of all epilepsy patients have epileptic seizures that are refractory to treatment [101]. At the moment, clinical trials in epilepsy focus mainly on the development of molecules that can prevent seizures in patients with drug-resistant epilepsy. Regulatory agencies have recently approved cenobamate, the first drug with a specific indication for refractory epilepsy, which has been shown to reduce seizure frequency in randomized, double-blind clinical trials [102]. This drug represented a turning point and has shed light on the development of new molecules that can contribute to the control of seizures in patients with refractory epilepsy.

However, all these drugs have been demonstrated to be effective agents in reducing the occurrence of seizures. An important distinction must be made between ictogenesis and epileptogenesis, which represent different physiopathological processes, and therefore their therapeutic targets should be different. Ictogenesis describes the processes of transition from the interictal state to a seizure, whereas epileptogenesis is the process by which a certain group or neuronal circuit becomes hyperexcitable, being able to spontaneously generate epileptic seizures. Advances in the knowledge of the genetics and pathophysiology of some specific diseases associated with epilepsy have led to the development of specific treatments for some syndromes, such as everolimus in tuberous sclerosis complex [103] or lysosomal enzyme replacement in neuronal ceroid lipofuscinosis [104]. Nonetheless, and particularly in adult-onset epilepsy, there are still many types of epilepsy and epileptic syndromes of which the specific etiopathogenesis is unknown, and therefore there are currently no specific therapeutic agents for those groups of patients. Interestingly, the potential bidirectional association of epilepsy and neurodegenerative processes opens the door to the development of new molecular targets that could potentially allow modifying the course of epilepsy.

Although some ASDs have been shown to have potential antiepileptogenic properties in animal models, such effects have not been confirmed in larger clinical studies [105]. In addition, a potential antiepileptogenic effect of several approved drugs, including atorvastatin, ceftriaxone, losartan, isoflurane, N-acetylcysteine, anakinra, rapamycin, and fingolimod, has been described in animal models [106,107,108,109,110,111,112]. Although the repositioning of these drugs could represent an attractive alternative in some specific etiologies, these results have not been confirmed by clinical trials [113]. This effect could be explained by the fact that most experimental studies on epileptogenesis have been strongly influenced by the kindling model, and the evidence supporting the existence of kindling in humans is controversial [114]. Most of these studies are based on post-traumatic or post-stroke epilepsy, which represents the archetype of epileptogenesis secondary to identifiable acquired brain injury. It is likely that the wide variety of etiologies, as well as the probably different mechanisms of epileptogenesis in other epilepsy syndromes, might have contributed to the difficulties in translating preclinical studies into clinical trials [113].

## 4. Antiseizure Drugs in Neurodegenerative Diseases

Because of the molecular links between epilepsy and other neurodegenerative diseases, various studies have been carried out to evaluate the therapeutic potential of anticonvulsant drugs in these pathologies and the therapeutic approach to epilepsy as a comorbidity. The wide variety of anticonvulsant drugs and their different mechanisms of action have positioned this group of drugs as very interesting candidates for those pathologies of the central nervous system with an uncertain origin or an inefficient available treatment. However, the potential neuroprotective role of these drugs in these pathologies remains unknown.

### 4.1. ASDs in Alzheimer’s Disease

In AD, some authors have tried to elucidate the pharmacological potential of ASDs in the pathological development of AD. For example, the research group of Dr. Mucke evaluated the effect of chronic treatment of levetiracetam (LEV) in the hAPP mice model, which has abnormally high amounts of human Aβ and displays abnormal neuronal network activity and epileptic seizures [115]. The authors found that LEV treatment was able not only to reduce abnormal spiking behavior and epileptiform discharges but also to suppress neuronal network dysfunction and reverse synaptic and cognitive deficits of these mice. Furthermore, several clinical trials aim to evaluate the effect of LEV in AD patients. For instance, a research group of the Johns Hopkins University Medical School conducted a Phase II trial to evaluate the effect of LEV on memory function in amnestic mild cognitive impairment (MCI) patients (NCT01044758). LEV was reported to reduce abnormal hyperactivity in the hippocampal dentate gyrus and CA3 regions, to boost abnormal hypoactivation in the entorhinal cortex, and to improve performance on the scanning memory task [116]. Similarly, other clinical trials are being conducted to evaluate LEV for the treatment of hyperexcitability and seizure activity in AD (NCT03875638, NCT03461861, NCT01554683) or to examine the effect of LEV on neuropsychiatric symptoms related to epilepsy (NCT04004702) [117]. In addition, a prospective, randomized, three-arm parallel-group, case-control study of AD patients taking LEV, phenobarbital, or lamotrigine showed that there were no significant differences in efficacy among these three ASDs, but LEV caused fewer adverse events than the other ASDs and was associated with improved cognitive performance and benign neuropsychological side effects [118]. Similarly, researchers of the Harvard Medical School carried out a feasibility study in which they evaluated the neurophysiological and cognitive effects of acute administration of LEV in patients with mild AD. They found that LEV positively alters the lower and higher frequency bands in the patients’ electroencephalogram, which represents the brain’s oscillatory connectivity. This suggests a beneficial effect of LEV for patients with AD [119]. Therefore, LEV is considered a cognitively safe ASD for AD patients. However, larger longitudinal studies, and studies with healthy age-matched controls, are needed to determine whether the effects of LEV are unique to AD as compared to normal aging and whether longer-term administration is associated with a beneficial clinical effect.

### 4.2. ASDs for Parkinson’s Disease

Dopamine agonists and levodopa for dopamine replacement are the current therapeutic approach for the treatment of PD. However, the effectiveness of these substances gradually diminishes, leading to an unstoppable progression of neurodegeneration. Because of that, many efforts have been made to find new or existing compounds that can be effective in PD. Some ASDs have been studied in this respect, and especially zonisamide (ZNS) has shown interesting results.

Several mechanisms have been proposed by which ZNS performs its beneficial effects: (i) inhibition of monoamine oxidase B, which reduces the dopamine-induced ROS production by the MAO-B pathway, thus contributing to nigrostriatal degeneration [120,121]; (ii) blocking of T-type calcium channels, resulting in an improvement in PD symptoms [122,123]; (iii) modulation of the levodopa-dopamine metabolism in the striatum by enhancing the dopamine synthesis and increasing the extracellular dopamine concentration [124]; (iv) downregulation of the expression of adenosine A2A and endocannabinoid CB1 receptors, which improves levodopa-induced dyskinesia [125]; and (v) neuroprotection, through the modulation of dopamine turnover, synaptic transmission, and gene expression and the induction of neurotrophic factors or the inhibition of neuroinflammation, oxidative stress, and apoptosis [126].

Many clinical trials have been carried out to explore the effectiveness of ZNS for the treatment of PD at different disease stages. In the early stages of the disease, an open-label clinical trial suggested that a single administration of ZNS improved motor and sleep dysfunction [127]. For advanced stages, several studies have evaluated the potential of ZNS as adjunctive therapy for motor fluctuations. Phase II and Phase III clinical trials demonstrated that ZNS improved motor functions and the wearing-off phenomenon without worsening dyskinesia in patients with advanced PD [128,129]. In the late stages of PD, only an open-label Phase II study was carried out. The obtained results showed that 300 mg/day of ZNS reduced the appearance of PD symptoms, especially those derived from the wearing-off phenomenon. The authors speculated that the long-lasting activation of dopamine synthesis by ZNS ameliorates PD symptoms, in particular the wearing-off phenomenon [130]. Nevertheless, the number of participants in this study was too low (n = 10) to draw definite conclusions, and further studies would be needed to validate all these findings. Currently, two clinical trials with ZNS are being developed to evaluate the role of ZNS in advanced PD (NCT04182399) and to examine the tolerability and efficacy of ZNS for dyskinesia in PD (NCT03034538). Preliminary results are not yet known.

### 4.3. ASDs for Huntington’s Disease

Since the symptomatology of HD is highly varied (chorea, dyskinesia, myoclonus, akathisia, bruxism, depression, cognitive and communication disorders, and memory deficits, among others), many drugs widely used in other pathologies have been explored in HD [131]. For example, ASDs have been the main candidates for treating myoclonus episodes. Myoclonus refers to sudden muscle contractions; they are brief and involuntary contractions similar to the spams and jerks of epileptic seizures but not related to epilepsy. In HD, myoclonus can be observed predominantly in juvenile forms but also in later-onset forms. Interestingly, in juvenile forms, non-epileptic myoclonus can coexist with epilepsy [131]. The use of valproate, alone or in combination with clonazepam, is recommended in these HD cases [131]. LEV is also recommended as a therapeutic alternative to valproate for the same indication. Likewise, the combination of valproate and olanzapine has been reported to help relieve agitation and aggression associated with HD [132]. When myoclonus has a cortical origin not associated with epileptic seizures, piracetam is authorized to be prescribed [132].

### 4.4. ASDs for Multiple Sclerosis

Patients with MS commonly suffer from neuropathic pain, which greatly affects their quality of life and which has a pooled prevalence of 63% [133]. ASDs are widely used to treat neuropathic pain in these patients. Antiepileptic drugs currently used for neuropathic pain are carbamazepine, oxcarbazepine, gabapentin, lacosamide, lamotrigine, clonazepam, levetiracetam, phenytoin, pregabalin, topiramate, and valproate. Nevertheless, the licensed status for this indication can vary in different countries [134]. In general, the hypothesis of the mechanism of action by which ASDs reduce neuropathic pain is based on their ability to reduce high-frequency neuronal firing. Three standard explanations have been described: (i) the inhibition of enhanced gamma-aminobutyric acid (GABA) (e.g., clonazepam or valproate), (ii) a stabilizing effect on neuronal cell membranes, possibly by modulating ion channels (e.g., gabapentin or lamotrigine), and (iii) the inhibition of NMDA receptor sites [134].

LEV has been shown to be effective not only in reducing neuropathic pain in MS patients but also in decreasing phasic spasticity. Hawker and colleagues performed a retrospective medical record review of patients attending the Multiple Sclerosis Program at the University of Texas. Their findings revealed that the Penn Spasm score (a measure of phasic spasticity) was decreased for all patients following treatment with LEV, and some patients also reported improvements in neuropathic pain [135]. Despite these promising results, large, well-controlled trials are needed to confirm these findings. Likewise, valproate has also been studied in a mice model of MS to evaluate its effectiveness in a variety of symptoms. The findings showed that valproate restored T-cell homeostasis and ameliorated the pathogenesis of these mice. However, further human studies should be performed to confirm these results [136].

Regarding clinical trials, completed studies have also evaluated the protective role of oxcarbazepine (NCT02104661) [137], lamotrigine (NCT00257855) [138], and LEV (NCT00423527) in MS patients. However, no consistent results have yet been obtained from these investigations. More studies with a larger sample size are needed to validate the evidence found so far.

## 5. Conclusions

Epilepsy affects approximately 50 million people worldwide. Developing countries are the most affected due to birth-related injuries, variations in medical infrastructure, and the low availability of preventive health programs. The massive entrance of Ca^2+^ into neurons is the main mechanism involved in the neuronal hyperexcitability that precedes seizures. However, many other mechanisms have been proposed to be associated with the development of seizures and epileptogenesis, and many of them are linked to those of major neurodegenerative diseases.

In AD, the role of Aβ peptides and p-tau in the development of neuroinflammation and neurodegeneration, as well as in the modulation of NMDA-Rs, AChRs, and ion channels, has been well described. All these alterations ultimately lead to the appearance of seizures. Similarly, the appearance of abnormal α-synuclein and mHtt in PD and HD, respectively, leads to mitochondrial damage that greatly affects the ionic balance in the neuron’s membrane. Likewise, an increase in oxidative stress, intracellular Ca^2+^, or proinflammatory cytokines also appears, contributing to aberrant neuronal hyperexcitability. In both PD and MS, a genetic correlation between them and epilepsy has not been found. However, many studies highlighted the appearance of seizures in these patients. In PD, a dual effect of dopamine related to seizure development has been shown. The activation of the D2 family of receptors triggers a protective pathway against seizure development, whereas the D1 family appears to activate a proepileptic pathway. In MS, the typical demyelination and axonal damage promoted by the autoimmune response also lead to an increased microglia response, elevated neurodegeneration, and, finally, increased neuronal excitability.

All these findings highlight the molecular cross-linking between epilepsy and major neurodegenerative diseases. The management of these alterations could open a promising window not only for epilepsy itself but also for epileptic comorbidities in other neurological diseases. Although many ASDs are available nowadays, a significant proportion of patients still have drug-resistant epilepsy. Because of that, several approved drugs have been studied in animal models for antiseizure applications, such as atorvastatin, ceftriaxone, losartan, anakinra, rapamycin, and fingolimod. Nevertheless, their potential use should be confirmed by clinical trials. Likewise, some commonly used ASDs, such as LEV, ZNS, and valproate, are being investigated in other neurodegenerative diseases, mainly because of the previously described molecular links and the lack of effective treatments for these diseases. Several clinical trials are being developed in this respect, but further studies are still needed to implement these therapies in clinical practice.

## Figures and Tables

**Table 1:** Main mechanisms of action of currently used ASDs.

Molecular Target		Antiseizure Drugs	Proposed Mechanisms of Action
**Voltage-gated ion channels**	Na^+^ channels	Phenytoin, fosphenytoin, carbamazepine, oxcarbazepine, eslicarbazepine acetate, lamotrigine, lacosamide, cenobamate *, rufinamide, topiramate, zonisamide	Enhancement of the rapid/slow inactivation of Na^+^ channels, inhibiting the propagation of action potentials
	Ca^2+^ channels	Ethosuximide	Inhibits hyperexcitability by regulating Ca^2+^ currents
	K^+^ channels	Retigabine (ezogabine)	Generates a subthreshold K^+^ current that stabilizes the membrane potential
**GABA-mediated inhibition**		Phenobarbital, primidone, benzodiazepines, stiripentol *, topiramate, felbamate, cenobamate, retigabine (ezogabine), tiagabine, vigabatrin, acetazolamide, topiramate, zonisamide, lacosamide *	Increased synaptic inhibition and reduced glutamate activity
**Synaptic release machinery**	SV2A	Levetiracetam, brivaracetam	Inhibition of excitatory neurotransmitter release
	α2δ subunit of voltage-gated Ca^2+^ channels	Gabapentin, pregabalin	Inhibition of excitatory neurotransmitter release
**AMPA receptor**		Perampanel	Inhibits the extracellular Ca^2+^ concentration and neuronal excitability
**Mixed/unknown**		Valproate, felbamate, cenobamate, topiramate, zonisamide, rufinamide, adrenocorticotrophin, cannabidiol	

Adapted from Sills and Rogawski (2020). * possible mechanism of action, not yet firmly established.

## Data Availability

Data sharing not applicable.

## Abbreviations

Aβ, amyloid-β; AD, Alzheimer’s disease; APP, amyloid precursor protein; ASDs, antiseizure drugs; BACE1, beta-secretase 1; BBB, blood-brain barrier; BDNF, brain-derived neurotrophic factor; CNS, central nervous system; GABA, gamma-aminobutyric acid; HD, Huntington’s disease; LEV, levetiracetam; mHtt, misfolded huntingtin protein; MS, multiple sclerosis; NFTs, neurofibrillary tangles; NMDA-R, N-methyl-D-aspartate receptor; PD, Parkinson’s disease; p-tau, phosphorylated tau; WHO, World Health Organization; ZNS, zonisamide.
